# Severity of COVID-19 in Cancer patients versus patients without Cancer: A Propensity Score Matching Analysis

**DOI:** 10.7150/jca.54205

**Published:** 2021-04-24

**Authors:** Chao Liu, Kai Wang, Luyuan Li, Qingquan Lv, Yumei Liu, Tian Hu, Jonathan C. Trent, Bing Sun, Qinyong Hu

**Affiliations:** 1 Department of Oncology, Renmin Hospital of Wuhan University, Wuhan, China.; 2 Department of Radiation Oncology, Shandong Cancer Hospital and Institute, Shandong First Medical University and Shandong Academy of Medical Sciences, Jinan, China.; 3 Department of Critical Care Medicine, Zhujiang Hospital, Southern Medical University, Guangzhou, China.; 4 Sylvester Comprehensive Cancer Center, University of Miami Miller School of Medicine, Miami, USA.; 5 Department of Medical Affairs, Wuhan Hankou Hospital, Wuhan, China.; 6 Department of Respiratory Medicine, Wuhan Hankou Hospital, Wuhan, China.; 7 Department of Radiation Oncology, the Fifth Medical Center, Chinese PLA General Hospital, Beijing, China.

**Keywords:** COVID-19, clinical characteristics, survival, propensity score matching, cancer

## Abstract

**Purpose:** Data are extremely limited with regards to the impact of COVID-19 on cancer patients. Our study explored the distinct clinical features of COVID-19 patients with cancer.

**Experimental Design:** 189 COVID-19 patients, including 16 cancer patients and 173 patients without cancer, were recruited. Propensity score 1:4 matching (PSM) was performed between cancer patients and patients without cancer based on age, gender and comorbidities. Survival was calculated by the Kaplan-Meier method and the difference was compared by the log-rank test.

**Results:** PSM analysis yielded 16 cancer patients and 64 propensity score-matched patients without cancer. Compared to patients without cancer, cancer patients tended to have leukopenia and elevated high-sensitivity C-reactive protein (hs-CRP) and procalcitonin. For those with critical COVID-19, cancer patients had an inferior survival than those without cancer. Also, cancer patients with severe/critical COVID-19 tended to be male and present with low S_P_O_2_ and albumin, and high hs-CRP, lactate dehydrogenase and blood urea nitrogen on admission compared to those with mild COVID-19. In terms of risk factors, recent cancer diagnosis (within 1 year of onset of COVID-19) and anti-tumor treatment within 3 months of COVID-19 diagnosis were associated with inferior survival.

**Conclusions:** We found COVID-19 patients with cancer have distinct clinical features as compared to patients without cancer. Importantly, cancer patients with critical COVID-19 were found to have poorer outcomes compared to those without cancer. In the cancer cohort, patients with severe/critical COVID-19 presented with a distinct clinical profile from those with mild COVID-19; short cancer history and recent anti-cancer treatment were associated with inferior survival.

## Introduction

The coronavirus disease 2019 (COVID-19) outbreak first emerged in December 2019, and has since spread globally. Subsequently, this resulted in a declaration of public health emergency of international concern on 30 January 2020 and was recognized as a pandemic on 11 March 2020. The clinical manifestation of COVID-19 range from asymptomatic infections to severe viral pneumonia, acute respiratory distress syndrome (ARDS) and death 1. A novel enveloped RNA coronavirus, now referred to as severe acute respiratory syndrome coronavirus 2 (SARS-Cov-2) has been identified as the causal pathogen 2. As of January 11, 2021, more than 88.3 million cases have been reported across 223 countries and territories, resulting in over 1.9 million deaths 3. SARS-CoV-2 associated lethality is mainly due to severe lower respiratory tract infections such as ARDS. This is similar to other two coronaviruses, SARS-CoV and MERS-CoV, the causal agents responsible for the severe acute respiratory syndrome (SARS; 2002-2003) and Middle East respiratory syndrome (MERS; 2012-ongoing) outbreaks, respectively 4. However, despite a lower case fatality rate (CFR), SARS-Cov-2 has proved more infectious than SARS-CoV and MERS-CoV, thus the overall number of deaths from COVID-19 far outweighs that from SARS or MERS 5.

Although COVID-19 is typically more severe and lethal among older people 6, people of any age with underlying medical conditions are at increased risk of COVID-19-associated adverse outcomes 7, 8. Cancer is an immunocompromised state, and cancer treatments can further compromise the immune system. As such, cancer patients are considered particularly susceptible to adverse outcomes from COVID-19. However, detailed data are extremely limited with regards to the impact of COVID-19 on cancer patients. To date, only few pertinent studies have been documented. Yu et al. reported a higher infection rate of SARS-CoV-2 in cancer patients and the association of infection with age and concurrent non-small cell lung carcinoma (NSCLC) 9. Zhang et al. observed that anti-tumor treatment within 14 days of infection and patchy consolidation on admission computed tomography (CT) are associated with poor outcomes 10. In addition, a prospective cohort study showed cancer patients have a higher risk of severe events relative to patients without cancer 11. However, due to limited clinical information or lack of appropriate controls, it is difficult to conduct more insightful analyses from these studies. In this study, we performed a propensity score matching analysis (PSM) among a total of 189 COVID-19 cases to eliminate the influence of potential confounding covariates across comparison groups. By analyzing 16 cancer patients and their propensity score-matched 64 patients without cancer, we revealed a panel of clinical features of SARS-CoV-2-infected patients in both cancer and non-cancer groups and in cancer subgroups (mild disease versus severe/critical disease), including demographic and clinical characteristics, radiological and laboratory findings as well as treatment and outcomes. Also, we investigated whether cancer treatment and duration of cancer history were associated with prognosis of COVID-19. Our study addressed the questions of which cancer patients, in terms of laboratory features, are going to likely develop severe/critical COVID-19, whether cancer patients with critical COVID-19 have worse outcomes than their controls without cancer, and what factors put cancer patients at high risk of poor outcomes from COVID-19.

## Methods

### Study design and participants

COVID-19 patients with or without cancer admitted in Wuhan Hankou Hospital between 5 January 2020 and 21 February 2020, were enrolled in this study. COVID-19 was diagnosed based on the criteria published by WHO 12 and confirmed by the detection of SARS-CoV-2 via real-time polymerase chain reaction (RT-PCR). To estimate the differences in prognosis and other clinical characteristics between patients with and without cancer, we performed PSM to select matched patients without cancer accounted for age, gender, and comorbidities which were recognized as potential risk factors of COVID-19 prognosis 13-15. Patients were matched 1:4 and randomly sorted using nearest neighbor technique with acceptable distance (caliper) of propensity scores. All patients had a definite clinical outcome (dead or discharged) by the final date of follow-up, 18 March 2020. This study was approved by the Ethical Committee of Wuhan Hankou Hospital (HKYY-2020-028), with a waiver of written informed consent.

### Data collection

Demographic, clinical, laboratory, and radiological data were retrieved from electronic medical records of Wuhan Hankou Hospital and reviewed independently by two physicians (KW and CL). Specifically, the data include patient-identified gender, age, symptoms, comorbidities, vital signs, coagulation and routine blood test, hepatic and renal function test, chest CT scans, treatments (antibiotic, antiviral, oxygen therapy, immunomodulators and glucocorticoids), and clinical outcomes (discharged or dead). Primary cancer characteristics (cancer type and history) and detailed treatment information (surgery, radiotherapy, chemotherapy, and targeted therapy) were obtained from patients' past medical records.

### Definition

The duration of COVID-19 was defined as the time interval between onset of symptoms and outcome (death or discharge from hospital). The clinical severity of COVID-19 was defined based on the criteria as described in the 6.0 version of Chinese management guideline for COVID-19, and patients were classified into mild (non-pneumonia and mild pneumonia), severe (S_P_O_2_ ≤ 93%, or respiratory frequency ≥ 30/min, or dyspnea, or lung infiltrates > 50% within 24 to 48 hours, or PaO_2_/FiO_2_ ≤ 300), or critical (multiple organ dysfunction or failure, or septic shock, or respiratory failure), accordingly.

### Statistical analysis

Categorical variables were presented as frequencies with percentages and compared by χ^2^ or Fisher's exact test. Laboratory findings, S_P_O_2_ (%), and respiratory and pulse rates were categorized based on the corresponding cut-off values for a normal range. Kaplan-Meier analysis and log-rank test were used to evaluated patients' survival and the differences between groups, respectively. All statistical analyses were performed using R (version 3.0.2) or SPSS 20.0 software (SPSS Inc, Chicago, IL), and a *p* value < .05 was considered statistically significant.

## Results

### Patient characteristics

We recruited a total of 189 patients with COVID-19 admitted in Wuhan Hankou Hospital between 5 January 2020 and 21 February 2020, comprising 16 cancer patients (CP) and 173 patients without cancer (PWC). Unbalanced covariates such as age, gender, and comorbidities were observed between CP and PWC groups. A propensity match was then performed, and 16 CP were matched 1:4 with 64 PWC. After matching, covariates were well balanced with no significant differences across two groups (Table 1).

Data including demographics, clinical characteristics, treatments and outcomes were shown in Table 1. Briefly, in the CP group, most were female (68.8%) and older than 60 (68.8%). The most common comorbidities were hypertension (56.3%), diabetes (25.0%) and coronary heart disease (18.8%). The most common presenting symptoms were fever (93.8%), cough (62.5%) and fatigue (56.3%). In terms of laboratory findings, patients most often had lower levels of albumin (56.3%), lymphocyte count (56.3%) and white blood cell count (43.8%), and higher levels of hs-CRP (87.5%), procalcitonin (75%) and lactate dehydrogenase (LDH) (62.5%). In terms of treatment, antibiotic therapy (68.8%) was most frequently administered, followed by antiviral therapy (68.8%) and immunomodulator (50%). In the PWC group, half were female (50.0%) and over half were older than 60 (62.5%). The top 3 common comorbidities were hypertension (43.8%), diabetes (15.6%) and coronary heart disease (12.5%). The top 3 presenting symptoms were fever (78.1%), cough (71.9%) and shortness of breath (37.5%). Patients most often had lower levels of albumin (57.8%), total protein (32.8%) and lymphocyte count (32.8%), and higher levels of hs-CRP (57.8%), procalcitonin (46.9%) and LDH (43.8%). Patients were most frequently given antibiotic therapy (84.4%), followed by antiviral therapy (46.9%) and glucocorticoid (46.9%).

### Comparison in clinical features between COVID-19 CP and PWC

With respect to symptoms on presentation, compared with PWC, CP tended to have fatigue and anorexia (56.3% *vs.* 31.3%, *p* = 0.083; 18.8% *vs.* 4.7%, *p* = 0.091, respectively), but the prevalence of other symptoms, including fever, cough, sputum, short of breath, diarrhea, nausea or vomiting, headache, myalgia, and sore throat was similar between two groups (all *p* > 0.05). Laboratory findings on admission showed CP tended to have leukopenia (43.8% *vs.* 15.6%, *p* = 0.035), lymphopenia (56.3% *vs.* 32.8%, *p* = 0.083), and elevated hs-CRP (87.5% *vs.* 57.8%, *p* = 0.027) and procalcitonin (75.0% *vs.* 46.9%, *p* = 0.044) than PWC (Table 1). There were no significant differences in other laboratory markers such as indicators of infection and hepatorenal function between CP and PWC. In terms of treatments, antiviral therapy appeared to be more frequently given to CP than PWC (68.8% *vs.* 46.9%, *p* = 0.164), but other treatments, including antibiotic therapy, immunomodulator, glucocorticoids, and oxygen therapy, were administered to patients with no preference (all *p* > 0.05).

### Comparison in COVID-19 clinical outcomes between CP and PWC

**Figure 1:**
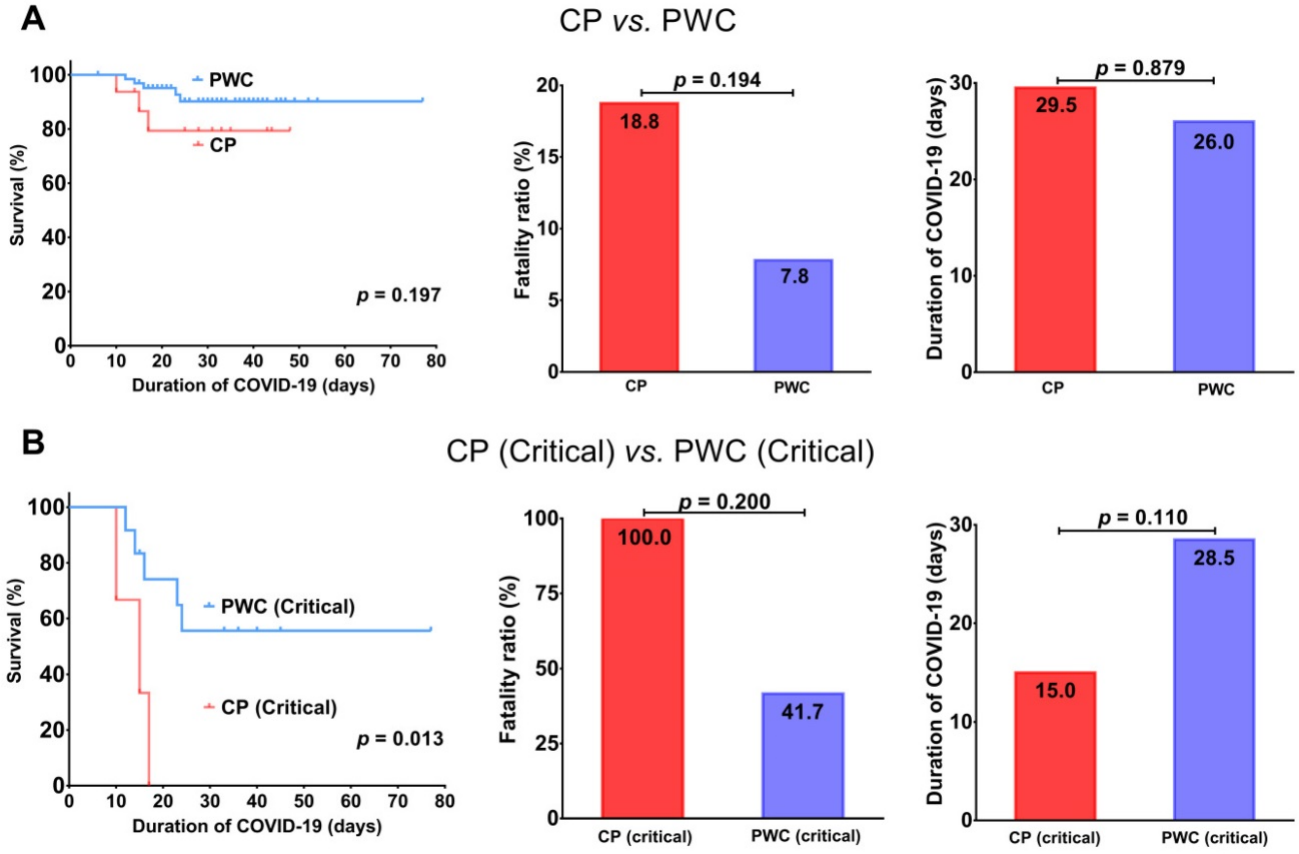
Comparisons of survival, fatality ratio and disease duration between COVID-19 CP and PWC (A); critical COVID-19 CP and PWC (B). CP, cancer patients; PWC, patients without cancer.

Overall, CP had a trend toward an inferior survival (*p* = 0.197) with a higher fatality ratio (18.8% *vs.* 7.8%, *p* = 0.194) and slightly longer disease duration (29.5 *vs.* 26.0 days, *p* = 0.879) than PWC (Figure 1A). Importantly, for patients with critical COVID-19, CP showed a significantly inferior survival (*p* = 0.013) with a trend toward a higher fatality ratio (100.0% *vs.* 41.7%, *p* = 0.200) and shorter disease duration compared to PWC (15.0 *vs.* 28.5 days, *p* = 0.110, Figure 1B), suggesting CP with critical COVID-19 tend to deteriorate rapidly and die during hospitalization.

**Figure 2:**
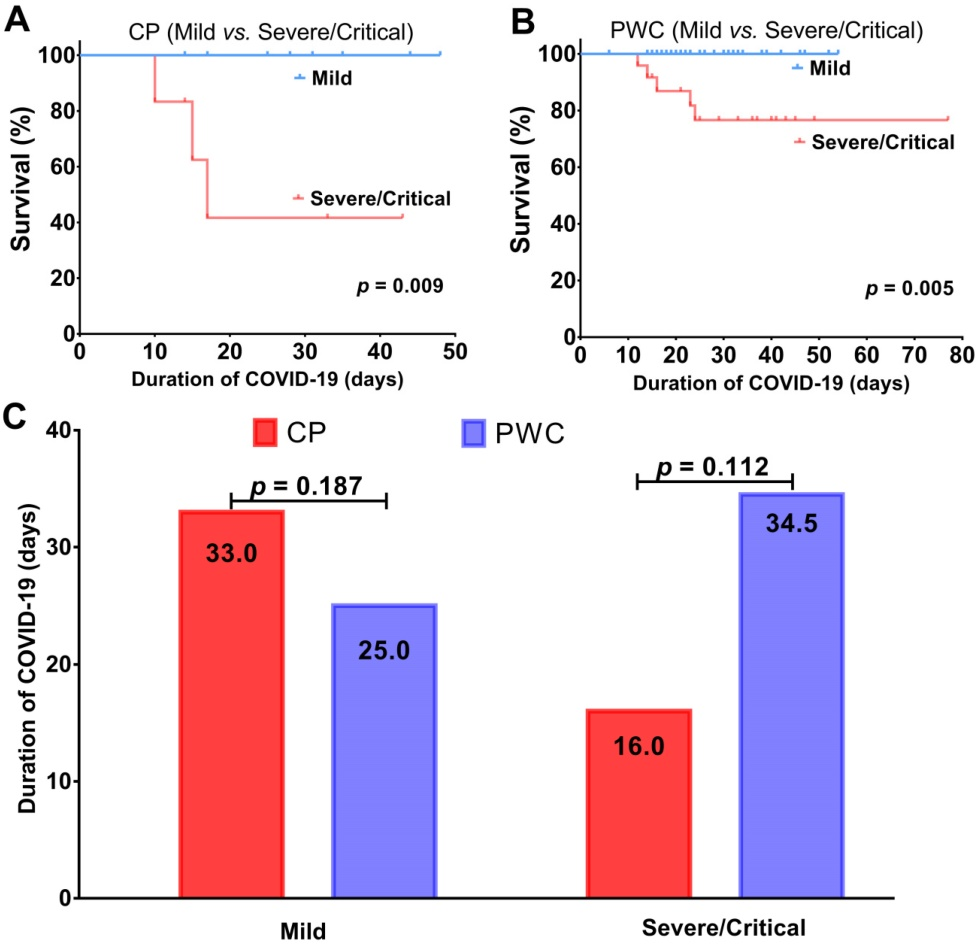
Survival analysis of mild and severe/critical COVID-19 CP (A); PWC (B); comparison of disease duration (C). CP, cancer patients; PWC, patients without cancer.

For patients with mild COVID-19, all were alive and discharged (Figure 2A-B), but CP showed a trend toward a longer disease duration than PWC (median, 33.0 *vs.* 25.0 days, *p* = 0.187, Figure 2C), suggesting CP with mild COVID-19 tend to have a longer recovery period. Consistent with the finding in patients with critical COVID-19, for patients with severe/critical COVID-19, CP had a trend toward a higher fatality ratio (50.0% *vs.* 20.8%, *p* = 0.30) with a shorter disease duration (median, 16.0 *vs.* 34.5 days, *p* = 0.112) than PWC (Figure 2C), suggesting CP with severe/critical COVID-19 tend to deteriorate rapidly and die during hospitalization. For patients with severe COVID-19, CP tend to have a slightly shorter disease duration (median, 33 *vs.* 38.5 days, *p* = 0.556) than PWC (Supplementary Figure 1).

### Comparison in clinical features between CP with mild and severe/critical COVID-19

According to the disease severity criteria for COVID-19 as described previously, among the 16 CP, 10 (62.5%) were classified into mild disease group while the rest 6 (37.5%) were classified into severe/critical disease group. The clinical characteristics of CP in both groups were shown in Table 2. Compared to those with mild disease, CP with severe/critical disease were significantly more likely to be male (66.7%* vs.*10%, *p* = 0.036) and present with abnormally low levels of S_P_O_2_ (83.3% *vs.* 0.0%, *p* = 0.001) and albumin (100.0% *vs.* 30.0%, *p* = 0.011), and also high levels of hs-CRP (100.0% *vs.* 40.0%, *p* = 0.034), LDH (83.3% *vs.* 20.0%, *p* = 0.035) and BUN (50.0% *vs.* 0.0%, *p* = 0.036) on admission. Although not statistically significant, there was a trend for the severe COVID-19 CP to be older than 60 (100.0% *vs.*50.0%, *p* = 0.059). Also, compared to those with mild disease, CP with severe/critical disease were more often given glucocorticoids (66.7 *vs.* 10.0%, *p* = 0.036), but no significant differences with respect to comorbidities, signs and symptoms were appreciated between two subgroups of CP. Moreover, as expected, CP with severe/critical disease had a higher fatality ratio (50.0% *vs.* 0.0%, *p* = 0.036, Table 2) and shorter disease duration (median, 16.0* vs.* 33.0 days, Figure 2C) than those with mild disease. The short disease duration of CP with severe/critical disease is attributed to the rapid patient deterioration during hospitalization. In addition, we also compared clinical features between PWC with mild and severe/critical COVID-19; details were shown in Supplementary Table 1.

### Tumor characteristics and risk factors for COVID-19 CP

**Figure 3:**
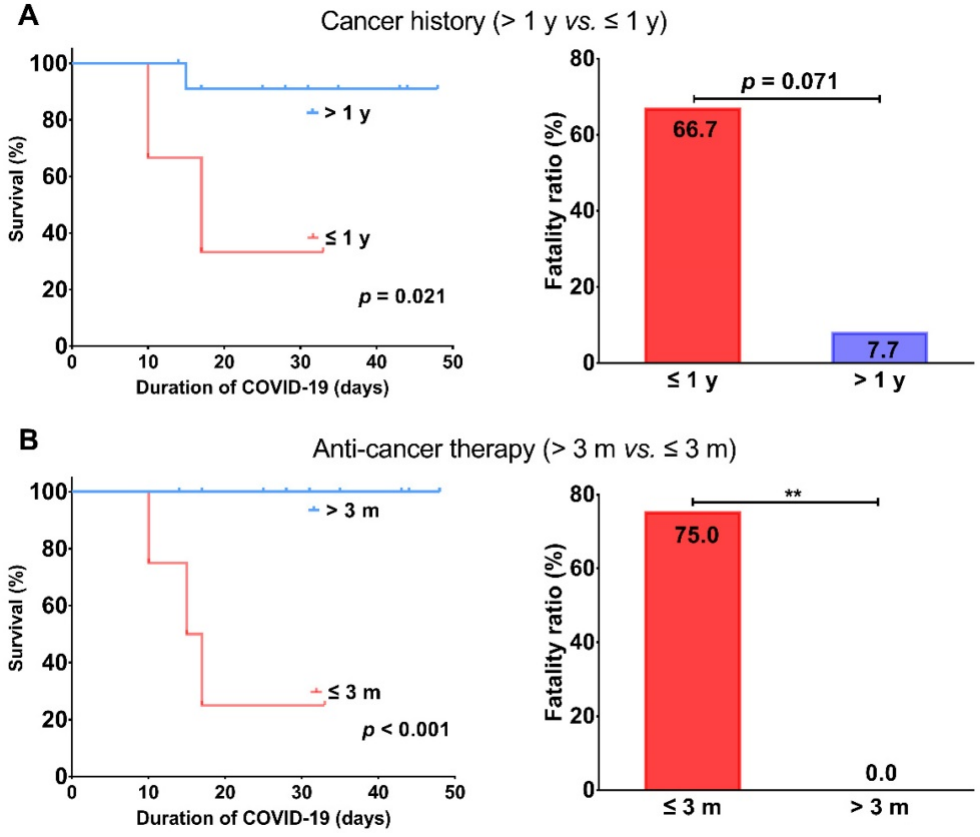
Comparisons of survival and fatality ratio between patients with cancer history > 1 year and ≤ 1 year (A) or anti-cancer therapy > 3 months and ≤ 3 months (B). ^**^ represents *p* < 0.01.

The primary tumor characteristics of 16 CP with COVID-19 were shown in Table 3. Colorectal cancer was the most frequent cancer type (5, 31.2%), followed by lung cancer (2, 12.5%), lymphoma (2, 12.5%), and cervical cancer (2, 12.5%) (Table 3). 13 (81.3%) patients were diagnosed with cancer > 1 year and 12 (75.0%) patients received antitumor treatment > 3 months prior to infection. The prevalence of severe/critical disease in patients with cancer history ≤ 1 year or anti-cancer treatment ≤ 3 months prior to infection was higher than those with cancer history >1 year or anti-tumor treatment >3 months prior to infection, respectively (*p* = 0.036, *p* = 0.003, respectively). In terms of outcome, patients with cancer history ≤ 1 year or anti-tumor treatment ≤ 3 months prior to infection had worse survival (*p* = 0.021, *p* < 0.001, respectively) with higher fatality ratios (66.7%* vs.* 7.7%, *p* = 0.071; 75% *vs.* 0%, *p* = 0.007, Figure 3) than patients with cancer history >1 year or anti-tumor treatment > 3 months prior to infection, respectively.

## Discussion

The rapidly expanding coronavirus pandemic has affected all areas of daily life, including medical care. Immunosuppression from malignant disease or its treatment renders cancer patients particularly susceptible to infections 16. Therefore, the clinicians need to weigh the risks of death and morbidity from the infection against the magnitude of benefit of intended cancer therapies. Our study aimed to understand the impact of COVID-19 on cancer patients, which will benefit the oncology society in guiding cancer care delivery amid this pandemic. We found that cancer patients with COVID-19 overall tend to have more specific onset symptoms such as anorexia and fatigue, leucopenia, lymphopenia, elevated hs-CRP and procalcitonin, longer disease duration and poorer outcomes compared to those without cancer. Importantly, we observed that cancer patients with critical disease have worse prognosis than their controls without cancer. Moreover, our study also showed short cancer history and recent anti-tumor treatments are associated with poor COVID-19 clinical outcomes.

Limited data suggested the likelihood of a severe illness from COVID-19 is higher among patients with cancer, particularly if they recently received or are continuing to receive treatment. Several studies were conducted on cancer patients diagnosed with COVID-19 from different countries, including the People's Republic of China, the United Kingdom, and the United States of America. These studies revealed that lung cancer, hematologic malignancy, lymphopenia, advanced age, male gender, and comorbidities were associated with greater severity of COVID-19 17-20. By analyzing 18 cancer patients from a nationwide cohort of 1590 COVID-19 cases, Liang et al. found compared with patients without cancer, cancer patients had a higher risk for severe clinical events (defined as invasive ventilation, ICU admission, or death) 11. However, in their study, the comparisons were directly made based on cancer patients and patients without cancer who had unbalanced potential confounding factors such as age, gender and comorbidities, posing a concern of biased estimate. To reduce the influence of these confounding factors, we performed PSM and made pairs from COVID-19 cancer patients and patients without cancer. By analyzing 16 cancer patients and their matched 64 non-cancer controls, we observed cancer patients overall have a relatively longer disease duration (29.5 *vs.* 26.0 days) and higher fatality ratio (18.8% *vs.* 7.8%). Noteworthy, the fatality ratios for both cancer patients and patients without cancer are much higher than the reported overall CFR, 2.3% or 5.6% among cancer patients 1. The high fatality ratios in our study can be attributed to the additive effects of other confounding factors such as age and pre-existing comorbid conditions (Table 1). Moreover, data show the CFR among critical cases of COVID-19 is dozens of times higher than overall 1. It is expected there is an exceptionally high fatality ratio among critical cases of cancer patients as well. Indeed, we observed up to half of the cancer patients with severe/critical disease in our study progressed rapidly and ended up with death during hospitalization. Interestingly, compared to those with mild disease, we noticed cancer patients with severe/critical disease tend to be male and older, and have low level of S_P_O_2_, high levels of hs-CPR and LDH, and abnormal levels of markers of poor hepatorenal function on admission (Table 2). We believe more distinctions between these two groups will be detected as sample size increases. Furthermore, given the quite different clinical characteristics of COVID-19 patients with mild and severe/critical COVID-19 7, 21, we further investigated the respective impact of COVID-19 on subgroups of cancer patients. Intriguingly, we observed cancer patients with mild disease present with similar clinical features to their controls without cancer except a longer disease duration which is indicative of slower recovery for cancer patients due to their compromised immune system. In contrast, cancer patients with critical disease have an inferior survival with a higher fatality ratio (100.0% *vs.* 41.7%) and shorter disease duration (15.0 *vs.* 28.5 days) compared to their controls without cancer, suggesting that cancer patients have a greater tendency to deteriorate fast and die from critical COVID-19. Our evidence here warrants an escalated level of care to this group of cancer patients.

Delivering cancer care is challenging during this COVID-19 crisis, and data pertaining to therapeutic perspective are urgently needed to help us weigh the competing risks of death or serious complications from cancer versus COVID-19. Zhang et al. retrospectively studied clinical features of 28 cancer patients with COVID-19. They found receiving anti-tumor treatment 14 days prior to infection significantly increased the risk of severe events (defined as the admission to ICU, or mechanical ventilation, or death) 10. However, their study is mainly based on severe cases, with over half of patients ending up with severe outcomes. Thus, their patient sample is not well representative of the whole cancer population with COVID-19. Given that 14-day is a relatively narrow interval which suits only a small portion of cancer patients, we extended this interval to 3-month in our study. Consistent with their study, we found patients receiving anti-cancer treatments (chemotherapy, surgery or targeted therapy) within 3 months prior to infection have an inferior survival with a higher fatality ratio than those receiving anti-cancer treatment more than 3 months prior to infection (75% *vs.* 0%). These findings suggest patients undergoing active cancer treatment have substantially increased risk of death from COVID-19. Thus, a delay of certain treatments will be more beneficial for patients with certain cancers that has low risk of progression in the midst of pandemic. We next explored whether long-term cancer survivors are also at increased risk of death from COVID-19. Our data demonstrated that patients with a cancer history less than 1 year have an inferior survival with a higher fatality ratio than those with a cancer history more than 1 year (66.7% *vs.* 7.7%). It should be noted the latter fatality ratio is close to that among patients without cancer (7.7% *vs.* 7.8%), indicating long-term cancer survivors may have a comparable risk of COVID-19 related lethality.

Through the use of COVID-19 patients without cancer as controls, novel and potentially useful clinical characteristics of cancer patients with COVID-19 were unveiled. By performing PSM, we balanced potential confounding covariates across groups of patients, and thus obtained an unbiased estimate of the impact of COVID-19 on cancer patients. In the future, it may be possible to identify cancer patients at risk for severe/critical COVID-19 using criteria including low SpO_2_ and albumin as well as high hs-CRP, LDH and BUN. Moreover, cancer patients diagnosed in the past year or treated with chemotherapy in the past 3 months and who may potentially be exposed to COVID-19 should be approached with strict PPE and physical distancing. However, this retrospective analysis has several limitations. This study was limited on a small sample size with heterogeneity such as various cancer types and COVID-19 treatment strategies. Due to the small sample size, some associations were inferred from the observed data trends instead of statistical significances. Thus, the key factors associated with severe/critical COVID-19 in cancer patients and other inferred associations should be verified in a larger study or a meta-analysis.

## Supplementary Material

Supplementary figures and tables.

## Figures and Tables

**Table 1:** Demographics, clinical characteristics, treatments and outcome of CP and PWC with COVID-19

Characteristic	Patients, No. (%)			*p* value^#^ (unmatched comparison)	*p* value^#^ (matched comparison)
	PWC before matching (n=173)	PWC after matching (n = 64)	CP (n = 16)		
Age, > 60y	89 (51.4)	40 (62.5)	11 (68.8)	0.185	0.642
Female	78 (45.1)	32 (50.0)	11 (68.8)	0.070	0.263
**Comorbidities**					
Coronary heart disease	12 (6.9)	8 (12.5)	3 (18.8)	0.120	0.685
Diabetes	24 (13.9)	10 (15.6)	4 (25.0)	0.264	0.463
Hypertension	59 (34.1)	28 (43.8)	9 (56.3)	0.045	0.412
Cerebrovascular disease	8 (4.6)	3 (4.7)	1 (6.3)	0.557	1.000
Chronic obstructive pulmonary disease	6 (3.5)	1 (1.6)	0 (0.0)	1.000	0.577
Chronic liver disease	4 (2.3)	2 (3.1)	1 (6.3)	0.361	0.493
Renal disease	5 (2.9)	3 (4.7)	1 (6.3)	0.416	1.000
**Signs and symptoms**					
Fever	142 (82.1)	50 (78.1)	15 (93.8)	0.316	0.281
Cough	125 (72.3)	46 (71.9)	10 (62.5)	0.399	0.545
Sputum	43 (24.9)	13 (20.3)	3 (18.8)	0.765	1.000
Shortness of breath	69 (39.9)	24 (37.5)	7 (43.8)	0.763	0.776
Diarrhea	13 (7.5)	4 (6.3)	0 (0.0)	0.607	0.579
Nausea or vomiting	4 (2.3)	1 (1.6)	0 (0.0)	1.000	1.000
Fatigue	62 (35.8)	20 (31.3)	9 (56.3)	0.107	0.083
Anorexia	23 (13.3)	3 (4.7)	3 (18.8)	0.466	0.091
Headache	3 (1.7)	1 (1.6)	0 (0.0)	1.000	1.000
Myalgia	4 (2.3)	3 (4.7)	2 (12.5)	0.083	0.260
Sore throat	4 (2.3)	2 (3.1)	1 (6.3)	0.361	0.493
Respiratory rate, > 20 times/min	64 (37.0)	17 (26.6)	6 (37.5)	0.968	0.537
Pulse rate, > 90 bpm	58 (33.5)	13 (20.3)	4 (25.0)	0.487	0.736
S_P_O_2_(%), < 93%	50 (28.9)	21 (32.8)	5 (31.3)	0.782	0.905
CO_2_ CP, > 29 mmol/L	20 (11.6)	10 (15.6)	2 (12.5)	1.000	1.000
CT findings (Bilateral)	149 (86.1)	56 (87.5)	15 (93.8)	0.700	0.679
**Laboratory Findings**					
White blood cell count, < 4×10^9^/L	35 (20.2)	10 (15.6)	7 (43.8)	0.053	0.035
Lymphocyte count, < 0.8×10^9^/L	64 (37.0)	21 (32.8)	9 (56.3)	0.130	0.083
Platelet count, < 100×10^9^/L	8 (4.6)	3 (4.7)	2 (12.5)	0.203	0.260
D-dimer, > 0.5 ug/ml	54 (31.2)	25 (39.1)	8 (50.0)	0.126	0.427
Hypersensitive C-reactive protein, > 6 mg/L	106 (61.3)	37 (57.8)	14 (87.5)	0.037	0.027
Procalcitonin, > 0.05 ng/mL	87 (50.3)	30 (46.9)	12 (75.0)	0.058	0.044
Lactate dehydrogenase, > 250 U/L	74 (42.8)	28 (43.8)	10 (62.5)	0.129	0.179
Alanine aminotransferase, > 40 U/L	36 (20.8)	8 (12.5)	3 (18.8)	1.000	0.685
Aspartate aminotransferase, >40 U/L	47 (27.2)	11 (17.2)	4 (25.0)	1.000	0.485
Alkaline phosphatase, > 140 U/L	6 (3.5)	2 (3.1)	0 (0.0)	1.000	1.000
r-glutamyl transferase, > 60 U/L	32 (18.5)	10 (15.6)	1 (6.3)	0.313	0.448
Total protein, < 60 g/L	52 (30.1)	21 (32.8)	6 (37.5)	0.575	0.723
Albumin, < 34 g/L	99 (57.2)	37 (57.8)	9 (56.3)	0.940	0.910
Globulin, < 26 g/L	32 (18.5)	12 (18.8)	2 (12.5)	0.741	0.724
Blood urea nitrogen, > 8.8 mmol/L	22 (12.7)	10 (15.6)	3 (18.8)	0.450	0.717
Creatinine, > 120 umol/L	18 (10.4)	6 (9.4)	3 (18.8)	0.395	0.373
Uric acid, > 440 umol/L	14 (8.1)	3 (4.7)	2 (12.5)	0.630	0.260
**Treatments**					
Antibiotic therapy	150 (86.7)	54 (84.4)	13 (81.3)	0.466	0.717
Antiviral therapy	86 (49.7)	30 (46.9)	11 (68.8)	0.145	0.164
Immunomodulator	91 (52.6)	29 (45.3)	8 (50.0)	0.842	0.785
Systemic glucocorticoids	98 (56.6)	30 (46.9)	5 (31.3)	0.051	0.399
Oxygen therapy	147 (85.0)	49 (76.6)	14 (87.5)	1.000	0.539
Nasal cannula	92 (53.2)	31 (48.4)	9 (56.3)	0.814	0.576
Mask oxygen	31 (17.9)	11 (17.2)	3 (18.7)	1.000	1.000
Mechanical ventilation	24 (13.9)	7 (10.9)	2 (12.5)	1.000	1.000
**Clinical outcome (died)**	22 (12.7)	5 (7.8)	3 (18.8)	0.450	0.194

Abbreviations: CP, cancer patients; PWC, patients without cancer. #Unmatched or matched comparison between PWC and CP.

**Table 2:** The differences of demographics, clinical characteristics, treatments and outcome between CP with mild and severe/critical COVID-19

Characteristic	CP, No. (%)		*p* value
	Mild (n = 10)	Severe/Critical (n = 6)	
Age, > 60y	5 (50.0)	6 (100.0)	0.059
Female	9 (90.0)	2 (33.3)	0.036
**Comorbidities**			
Coronary heart disease	3 (30.0)	1 (16.7)	1.000
Diabetes	2 (20.0)	2 (33.3)	0.604
Hypertension	6 (60.0)	3 (50.0)	1.000
Cerebrovascular disease	1 (10.0)	0 (0.0)	1.000
Chronic obstructive pulmonary disease	0 (0.0)	0 (0.0)	/
Chronic liver disease	0 (0.0)	1 (16.7)	0.375
Renal disease	1 (10.0)	0 (0.0)	1.000
**Signs and symptoms**			
Fever	9 (90.0)	6 (100.0)	1.000
Cough	7 (70.0)	3 (50.0)	0.607
Sputum	2 (20.0)	1 (16.7)	1.000
Shortness of breath	4 (40.0)	3 (50.0)	1.000
Diarrhea	0 (0.0)	0 (0.0)	/
Nausea or vomiting	0 (0.0)	0 (0.0)	/
Fatigue	5 (50.0)	4 (66.7)	0.633
Anorexia	1 (10.0)	2 (33.3)	0.518
Headache	0 (0.0)	0 (0.0)	/
Myalgia	2 (20.0)	0 (0.0)	0.500
Sore throat	0 (0.0)	1 (16.7)	0.375
Respiratory rate, > 20 times/min	4 (40.0)	2 (33.3)	1.000
Pulse rate, > 90 bpm	3 (30.0)	1 (16.7)	1.000
S_P_O2(%), < 93%	0 (0.0)	5 (83.3)	0.001
CO2 CP, > 29 mmol/L	2 (20.0)	0 (0.0)	0.500
CT findings (Bilateral)	9 (90.0)	6 (100.0)	1.000
**Laboratory Findings**			
White blood cell count, < 4×10^9^/L	5 (50.0)	2 (33.3)	0.633
Lymphocyte count, < 0.8×10^9^/L	4 (40.0)	5 (83.3)	0.145
Platelet count, < 100×10^9^/L	1 (10.0)	1 (16.7)	1.000
D-dimer, > 0.5 ug/ml	3 (30.0)	4 (66.7)	0.302
Hypersensitive C-reactive protein, > 6 mg/L	4 (40.0)	6 (100.0)	0.034
Procalcitonin, > 0.05 ng/mL	6 (60.0)	5 (83.3)	0.588
Lactate dehydrogenase, > 250 U/L	2 (20.0)	5 (83.3)	0.035
Alanine aminotransferase, > 40 U/L	1 (10.0)	2 (33.3)	0.518
Aspartate aminotransferase, >40 U/L	2 (20.0)	2 (33.3)	0.604
Alkaline phosphatase, > 140 U/L	0 (0.0)	0 (0.0)	/
r-glutamyl transferase, > 60 U/L	0 (0.0)	2 (33.3)	0.125
Total protein, < 60 g/L	3 (30.0)	3 (50.0)	0.607
Albumin, < 34 g/L	3 (30.0)	6 (100.0)	0.011
Globulin, < 26 g/L	1 (10.0)	0 (0.0)	1.000
Blood urea nitrogen, > 9.5 mmol/L	0 (0.0)	3 (50.0)	0.036
Creatinine, > 120 umol/L	1 (10.0)	2 (33.3)	0.518
Uric acid, > 440 umol/L	1 (10.0)	1 (16.7)	1.000
**Treatments**			
Antibiotic therapy	7 (70.0)	6 (100.0)	0.250
Antiviral therapy	6 (60.0)	5 (83.3)	0.588
Immunomodulator	5 (50.0)	3 (50.0)	1.000
Systemic glucocorticoids	1 (10.0)	4 (66.7)	0.036
Oxygen therapy	8 (80.0)	6 (100.0)	0.500
Nasal cannula	7 (70.0)	2 (33.3)	0.362
Mask oxygen	1 (10.0)	2 (33.3)	0.620
Mechanical ventilation	0 (0.0)	2 (33.3)	0.125
**Clinical outcome (died)**	0 (0.0)	3 (50.0)	0.036

Abbreviations: CP, cancer patients.

**Table 3:** Cancer characteristics in 16 COVID-19 CP

Characteristic	CP, No. (%)				*p* value
	Total (n = 16)	Mild (n = 10)	Severe (n = 3)	Critical (n = 3)	
**Cancer type**					
Nasopharyngeal cancer	1 (6.3)	1 (10.0)	0 (0.0)	0 (0.0)	0.955
Thyroid cancer	1 (6.3)	1 (10.0)	0 (0.0)	0 (0.0)	
Breast cancer	1 (6.3)	1 (10.0)	0 (0.0)	0 (0.0)	
Lung cancer	2 (12.5)	1 (10.0)	1 (33.3)	0 (0.0)	
Colorectal cancer	5 (31.3)	2 (20.0)	2 (66.7)	1 (33.3)	
Gastric cancer	1 (6.3)	0 (0.0)	0 (0.0)	1 (33.3)	
Lymphoma	2 (12.5)	1 (10.0)	0 (0.0)	1 (33.3)	
Cervical cancer	2 (12.5)	2 (20.0)	0 (0.0)	0 (0.0)	
Skin cancer	1 (6.3)	1 (10.0)	0 (0.0)	0 (0.0)	
**Cancer history**					
> 1y	13 (81.3)	10(100.0)	2 (66.7)	1 (33.3)	0.036
≤ 1y	3 (18.8)	0 (0.0)	1 (33.3)	2 (66.7)	
**Cancer therapy prior to COVID-19**					
> 3m	12 (75.0)	10(100.0)	2 (66.7)	0 (0.0)	0.003
≤ 3m	4 (25.0)	0 (0.0)	1 (33.3)	3 (100.0)	
**Cancer therapy within 3 months**					
Surgery and chemotherapy	1 (6.3)	0 (0.0)	0 (0.0)	1 (33.3)	1.000
Chemotherapy	2 (12.5)	0 (0.0)	1 (33.3)	1 (33.3)	
Targeted therapy	1 (6.3)	0 (0.0)	0 (0.0)	1 (33.3)	

Abbreviations: CP, cancer patients.
