# Supplementary material for: Severity of COVID-19 in Cancer patients versus patients without Cancer: A Propensity Score Matching Analysis
Source: J Cancer. 2021 Apr 24;12(12):3558–65. doi: 10.7150/jca.54205 (PMC8120166; doi:10.7150/jca.54205)
Supplement: Supplementary file 1 — Supplementary figures and tables. [file jcav12p3558s1.pdf]

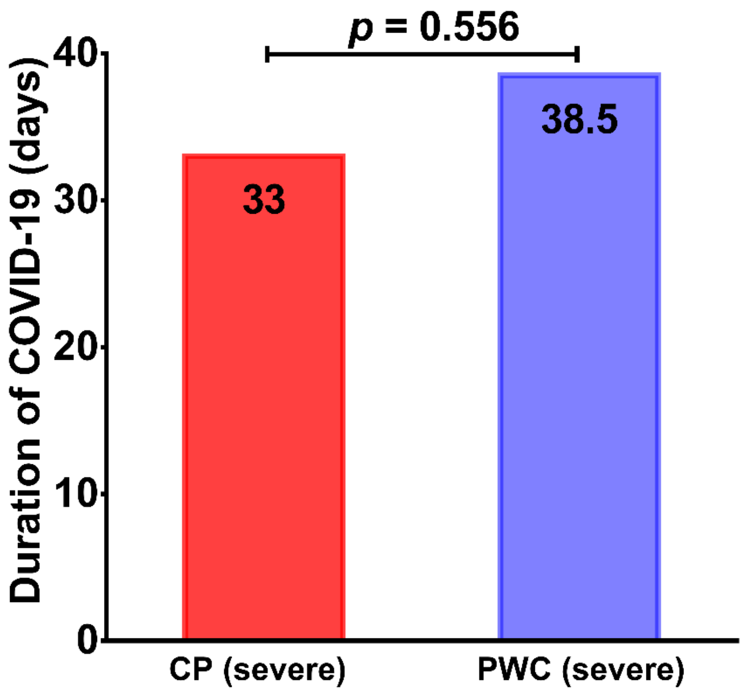

Supplementary Figure 1. Comparison of disease duration between CP and PWC with severe COVID-19.

Supplementary Table 1. The differences of demographics, clinical characteristics, treatments and outcome between PWC with mild and severe/critical COVID-19

**Characteristic**	PWC, No. (%)		
	Mild (n = 40)	Severe/Critical (n = 24)	*p* value
Age, > 60y	17 (42.5)	23 (95.8)	< 0.001
Female	24 (60.0)	8 (33.3)	0.039
**Comorbidities**			
Coronary heart disease	2 (5.0)	3 (12.5)	0.548
Diabetes	4 (10.0)	5 (20.8)	0.403
Hypertension	15 (37.5)	13 (54.2)	0.193
Cerebrovascular disease	2 (5.0)	2 (8.3)	1.000
Chronic obstructive pulmonary disease	3 (7.5)	2 (8.3)	1.000
Chronic liver disease	2 (5.0)	0 (0)	0.524

Renal disease	2 (5.0)	1 (4.2)	1.000
**Signs and symptoms**			
Fever	30 (75.0)	20 (83.3)	0.639
Cough	26 (65.0)	20 (83.3)	0.196
Sputum	7 (17.5)	6 (25.0)	0.470
Shortness of breath	12 (30.0)	12 (50.0)	0.110
Diarrhea	0 (0.0)	4(16.7)	0.017
Nausea or vomiting	1 (2.5)	0 (0.0)	1.000
Fatigue	12 (30.0)	8 (33.3)	0.781
Anorexia	1(2.5)	2 (8.3)	0.551
Headache	1(2.5)	0 (0.0)	1.000
Myalgia	2 (5.0)	1 (4.2)	1.000
Sore throat	2 (5.0)	0 (0.0)	0.524
Respiratory rate, > 20 times/min	10 (25.0)	7 (29.2)	0.715
Pulse rate, > 90 bpm	6 (15.0)	7 (29.2)	0.173
S_PO_2(%), < 93%	8 (20.0)	13 (54.2)	0.005
CO_2 CP, > 29 mmol/L	9 (22.5)	1 (4.2)	0.110
CT findings (Bilateral)	36(90.0)	20 (83.3)	0.435
**Laboratory Findings**			
White blood cell count, < 4×10^9/L	9 (22.5)	1 (4.2)	0.110
Lymphocyte count, < 0.8×10^9/L	8 (20.0)	13 (54.2)	0.005
Platelet count, < 100×10^9/L	0 (0)	3 (12.5)	0.049
D-dimer, > 0.5 ug/ml	12 (30.0)	7 (29.2)	0.994
Hypersensitive C-reactive protein, > 6 mg/L	16 (40.0)	12 (50.0)	0.435
Procalcitonin, > 0.05 ng/mL	10 (25.0)	12 (50.0)	0.041
Lactate dehydrogenase, > 250 U/L	11 (27.5)	15 (62.5)	0.006
Alanine aminotransferase, > 40 U/L	4 (10.0)	4 (16.7)	0.696
Aspartate aminotransferase, >40 U/L	5 (12.5)	6 (25.0)	0.199

Alkaline phosphatase, > 140 U/L	2 (5.0)	0 (0.0)	0.711
r-glutamyl transferase, > 60 U/L	4 (10.0)	6 (25.0)	0.213
Total protein, < 60 g/L	13 (32.5)	8 (33.3)	0.945
Albumin, < 34 g/L	19 (47.5)	17 (70.8)	0.069
Globulin, < 26 g/L	10 (25.0)	2 (8.3)	0.186
Blood urea nitrogen, > 9.5 mmol/L	2 (5.0)	7 (29.2)	0.020
Creatinine, > 120 umol/L	4 (10.0)	2 (8.3)	1.000
Uric acid, > 440 umol/L	1 (2.5)	2 (8.3)	0.647
**Treatments**			
Antibiotic therapy	31 (77.5)	23 (98.8)	0.051
Antiviral therapy	21(52.5)	9 (37.5)	0.244
Immunomodulator	13 (32.5)	15 (62.5)	0.019
Systemic glucocorticoids	13 (32.5)	17 (70.8)	0.003
Oxygen therapy	25 (62.5)	24 (100.0)	0.001
Nasal cannula	24 (60.0)	7 (29.1)	0.017
Mask oxygen	1 (2.5)	10 (41.7)	< 0.001
Mechanical ventilation	0 (0.0)	7 (29.2)	0.001
**Clinical outcome (died)**	0 (0.0)	5 (20.8)	0.006

Abbreviations: PWC, patients without cancer
